# Time-Dependent Alterations in Rat Macrovessels with Type 1 Diabetes

**DOI:** 10.1155/2012/278620

**Published:** 2012-01-23

**Authors:** Yvonne Searls, Irina V. Smirnova, Lisa VanHoose, Barbara Fegley, Rajprasad Loganathan, Lisa Stehno-Bittel

**Affiliations:** ^1^Department of Physical Therapy and Rehabilitation Science, University of Kansas Medical Center, Kansas City, KS 66160, USA; ^2^Department of Anatomy and Cell Biology, University of Kansas Medical Center, Kansas City, KS 66160, USA

## Abstract

Vascular complications are associated with the progressive severity of diabetes, resulting in significant morbidity and mortality. This study quantifies functional vascular parameters and macrovascular structure in a rat model of type 1 diabetes. While there was no difference in the systemic arterial elastance (Ea) with 50 days of diabetes, changes were noted in the aorta and femoral artery including increased tunica media extracellular matrix content, decreased width of both the media and individual smooth muscle cell layers, and increased incidence of damaged mitochondria. Extracellular matrix proteins and elastin levels were significantly greater in the aorta of diabetic animals. These differences correlated with diminished matrix metalloprotease activity in the aorta of the diabetic animals. In conclusion, diabetes significantly altered the structure and ultrastructure of the aorta and femoral artery before systemic changes in arterial elastance could be detected.

## 1. Introduction

The worldwide incidence of type 1 diabetes is increasing at a rate of 3% per year [1]. Macrovascular disease, affecting large vessels, is still the principal cause of morbidity and mortality for people with diabetes [2], even as new treatments have greatly decreased the incidence of microvascular complications associated with retinopathy, nephropathy, and neuropathy. Since type 1 diabetes is typically diagnosed in youth and young adults, macrovascular changes occur early [3] and progress throughout the person's life [4]. In people with diabetes, the ability of the macrovessels to respond to agonists decreases with age [5].

Clinical tools used to assess large vessel pathology indicate that vessel wall structure and stiffness change early in the progression of diabetes [6]. These clinical tools include measurement of the intima-media thickness to determine structural changes while pulse wave velocity assesses arterial stiffness. Changes in both parameters occur early in the progression of type 1 diabetes and tend to become more severe with the duration of the disease [7, 8]. Stiffness changes associated with diabetes predominantly affect the aorta and the proximal arteries. Accordingly, the carotid-aorto-femoral pulse wave velocity was highly correlated with the duration of type 1 diabetes in humans [9]. This loss of vascular compliance is a powerful predictor of cardiovascular and all-cause mortality for people with diabetes [10].

A change in arterial mechanical properties can occur with increased collagen cross-linking as a result of glycation [11, 12]. In humans, the accumulation of certain advanced glycation end products increases the likelihood of developing diabetic complications [13]. Diabetes-induced ultrastructural changes in streptozotocin-(STZ-) treated animal models include increased accumulation of extracellular matrix (ECM) proteins, migration of smooth muscle cells (SMCs), irregular SMC cytoplasm, and endothelial alterations [14], which model well the morphological changes observed in human veins [15].

 The purpose of this study was to determine the morphometric characteristics of diabetes-induced abnormalities of two major vessels, the aorta and the femoral artery, at an early time point before functional changes were noted at the systemic level and follow those changes as the disease progressed.

## 2. Methods and Materials

### 2.1. Animals

Thirty-six male Sprague Dawley rats (Harlan, Indianapolis, IN) weighing 250–270 grams were divided into two groups: diabetic and nondiabetic control (*n* = 18 diabetic and *n* = 18 control). Diabetes was induced with intraperitoneal injections of streptozotocin (65 mg/kg, Sigma-Aldrich, St. Louis, MO). Control rats were injected with a vehicle (citrate buffer). 

Development of diabetes was determined by observing blood glucose levels greater than 300 mg/dL as measured by an Accu-Check Advantage glucometer (Boehringer Mannheim Corporation, Indianapolis, IN). Rat body weights and blood glucose levels were recorded weekly. Rats were provided free access to food and water, and principles of institutional laboratory animal care were strictly followed. The rats were sacrificed with an intraperitoneal injection of pentobarbital.

### 2.2. Vascular Resistance

Functional evaluation was performed using left ventricular catheterization through the right carotid artery with a 2 French Millar microtip pressure volume catheter (Millar Instruments, Houston, TX) as previously described [16], under ketamine and xylazine anesthesia (80 mg/Kg and 10 mg/Kg, resp.). Heart rate was monitored during the procedure. After allowing the pressure volume (PV) loops to stabilize for 3–5 minutes, steady-state PV loops were recorded for 1 minute at a sampling rate of 1000 samples per second using Millar Pressure Volume System (MPVS-400, AD Instruments, Colorado Springs, CO). On average, 5-6 loops per animal were used for analysis of arterial elastance using PVAN 3.3 software (ADInstruments, Colorado Springs, CO). Calibration of the pressure volume catheter was performed per manufacturer's instructions with hypertonic saline and fresh heparinized rat blood. A blood sample was taken from each animal group to calculate the formula for volume calibration.

### 2.3. Tissue Procurement

Following animal sacrifice, one-to-two millimeter segments of thoracic aorta and proximal sections of the femoral artery were immediately resected and rinsed in ice-cold phosphate-buffered saline. Samples were gathered from the center sections of the vessels and quickly immersed in fixative at +4°C.

### 2.4. Light Microscopy Studies

Samples of thoracic aorta and femoral artery were fixed in 4% paraformaldehyde and stored at +4°C. Tissues were processed by rinsing with water before undergoing dehydration through a graded ethanol series ending in xylene. Samples were embedded in paraffin. Five micrometer paraffin sections were cut and stained with hematoxylin and eosin (H&E) or Masson's trichrome. Vessels from three rats in each group were used for calculations. Digital images were acquired using a Nikon Eclipse TE300 microscope attached to a SPOT 32 system (Diagnostic Instruments, Inc., Sterling Heights, MI).

All analyses were blinded and completed on digital images using Adobe Photoshop (Adobe Systems Inc.) or Scion Image (Scion Corporation). Vessel width was measured at 10 evenly spaced points along the vessel at 20x. All other analyses were completed on images taken at 40x magnification. The SMC layer width was measured at 10 evenly spaced sites between elastic bands along the vessel length of all but the first and last layers within the media to eliminate any sectioning artifacts or subsequent misidentification of endothelium or adventitia. Total vessel area measurement including intima, media, and adventitia and arterial ECM content was calculated by color analysis from sections stained with Masson's trichrome.

### 2.5. Electron Microscopy Studies

Tissues were fixed in 2% glutaraldehyde, rinsed in PBS and postfixed with 1% osmium tetroxide. Rinsing with distilled water was followed with a graded ethanol dehydration series ending with propylene oxide. The samples were infiltrated using a mixture of 1/2 propylene oxide and 1/2 resin overnight and then embedded in Epon 812 resin (Electron Microscopy Sciences, Ft. Washington, PA). One-micron-thick sections of the vessel were examined under the light microscope before 80 nm sections were cut on an LKB Nova Ultratome. Thin sections were captured on precleaned copper grids and stained using a double lead stain technique [17] with 0.5% lead citrate and 7% uranyl acetate. Images were captured from random tissue sections using a JEM 100 CXII transmission electron microscope at 80 KV.

### 2.6. Morphometric Analysis

Lipid incidence, mitochondrial area, and mitochondrial membrane integrity were assessed from electron microscopy (EM) photomicrographs taken at final magnifications of 8,700x and 21,600x. To assess mitochondrial quality, each mitochondrion was graded as intact or compromised using previously published techniques [18]. This grading system scores the mitochondrial membranes as completely intact, disrupted outer membrane, disrupted inner membrane, or disruption of both mitochondrial membranes. The mitochondrial scoring was completed in a blinded fashion. Analysis of the nuclei was done at final magnification of 21,600x.

### 2.7. Matrix Metalloprotease (MMP) Zymography

Aorta was removed from a STZ-injected rat at 50 days of diabetes and ~8 mm piece was cut and rinsed in ice cold 0.15 M NaCl. The tissue was homogenized using Polytron homogenizer in an ice-cold extraction buffer (1 : 3 wt/vol) containing the following: 50 mM Tris-HCl, pH 7.5, 0.15 M NaCl, 10 mM CaCl_2_, 0.05% Brij-35, and 0.02% NaN_3_. The homogenate were shaken on cold overnight, then centrifuged at 4°C for 30 min at 12,000 ×g and the supernatants were collected. Protein concentration was measured using Protein Assay Reagent (Bio-Rad, Hercules, CA).

In [19, 20] Quantitative MMP zymography was performed using previously described procedure. Ten % polyacrylamide gel was prepared containing mg/mL gelatin (Fisher Scientific, Pittsburg, PA). Aorta extracts (10 *μ*g total protein) were mixed with sample loading sodium dodecyl sulfate (SDS) buffer without beta-mercaptoethanol and heating, loaded on the gel, and proteins were separated electrophoretically. After electrophoresis, the gel was agitated in the washing buffer, containing 50 mM Tris-HCl, pH 7.5, 5 mM CaCl_2_, 1 *μ*M ZnSO_4_, 0.02% NaN_3_, and 2.5% Triton X-100, for 30 min twice at room temperature, to remove SDS. Subsequently, the gel was kept in the incubation buffer, containing 50 mM Tris-HCl, pH 7.5, 5 mM CaCl_2_, 1 *μ*M ZnSO_4_, and 0.02% NaN_3_, overnight at 37°C to allow MMPs to cleave gelatin within the gel. Finally, the gel was stained with Coomassie Brilliant Blue 250 while agitating for 1 h and destained in water until clear bands were visible. The intensity of the bands was measured in a negative mode using Adobe Photoshop and MMP activity was expressed in arbitrary units. As positive control for zymography, purified commercial bacterial collagenase (Gibco BRL, Rockville, MD) was used.

### 2.8. Data Analysis

Quantitative analysis of the ultrastructure was completed on photomicrographs taken from a minimum of five independent regions within a section from each sample at each magnification aforementioned. Statistical analysis using Sigma Stat software (SPSS, Inc.) employed *t*-tests for comparison of diabetic and control groups and one way ANOVA for analysis within groups. When the data did not meet normality requirements, a Mann-Whitney Rank Sum test was employed. All bar graphs illustrate mean ± SE. *P* value ≤ 0.05 was considered statistically significant.

## 3. Results

### 3.1. Animal Gravimetry and Glucometry

The body weight of the STZ-diabetic rats and matched controls was not different at the initiation of the study. The control animals weighed 257.7 ± 2.3 gm, while the animals assigned to the diabetic group weighed 262.2 ± 2.8 gm. Over time, both groups of animals gained weight, but the diabetic group gained less weight than the controls. At 50 days, the control animals weighed 452.9 ± 15.8 gm and the diabetic rats weighed 327.7 ± 14.4 gm, which was significantly different (*P* < 0.05). 

Nonfasting blood glucose levels, while between 101–113 mg/dL in control rats, increased 471% with 50 days of diabetes, 509% with 100 days of diabetes, and 407% with 150 days of diabetes. The high glucose levels in the diabetic rats were consistent across the weekly measurements. The average blood glucose levels at the time of termination are presented in Table 1.

### 3.2. Vascular Physiology

**Figure 1:**
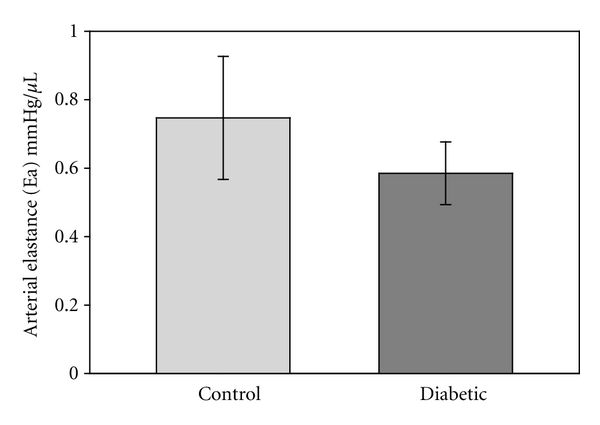
Arterial elastance was measured via catheters placed within the left ventricle. No difference was detected between the control and diabetic groups (*n* = 6/group).

There was no statistical difference in resting blood pressure recordings, obtained under anesthesia, for the diabetic group compared to the matched controls. With 50 days of diabetes the mean blood pressure of the control group was 109.7 ± 2.4 mmHg, while the mean blood pressure of the diabetic group was 105.9 ± 2.1 mmHg (*n* = 6/group). Blood pressure measurements were not taken on the 100 or 150 day diabetic animals. There was also no significant difference in average heart rates between groups: controls levels were 183 ± 33 bpm, while the diabetic group averaged 205 ± 27 bpm (*n* = 7/group). Arterial elastance, a standard measure of arterial impedance, and more specifically aortic impedance [16], was determined from PV loops obtained from the left ventricle and showed no difference between the groups (Figure 1).

### 3.3. Vascular Structure

**Figure 2:**
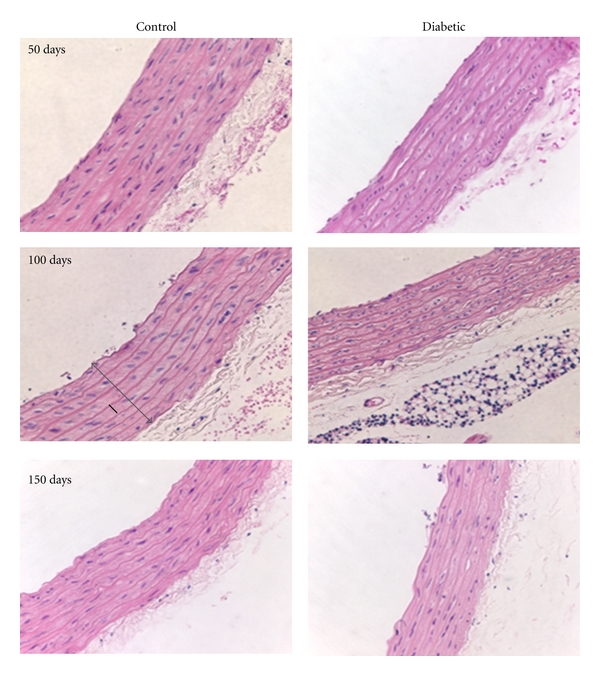
Morphological duration-dependent changes in the aorta of diabetic rats. Control aorta showed a typical H&E staining pattern with SMCs and elastic layers staining pink (lighter and darker, resp.) while nuclei were purple. At the 150-day time point, the SMC layers became slightly less defined and the vessel wall was reduced in cross-sectional area. The measured vessel wall width (tunica intima-media width) is illustrated by the gray arrow in the 100-day control image. An example of the SMC layer width measurements is shown with a solid black line in the 100 day control image (Magnification: 40x).

**Figure 3:**
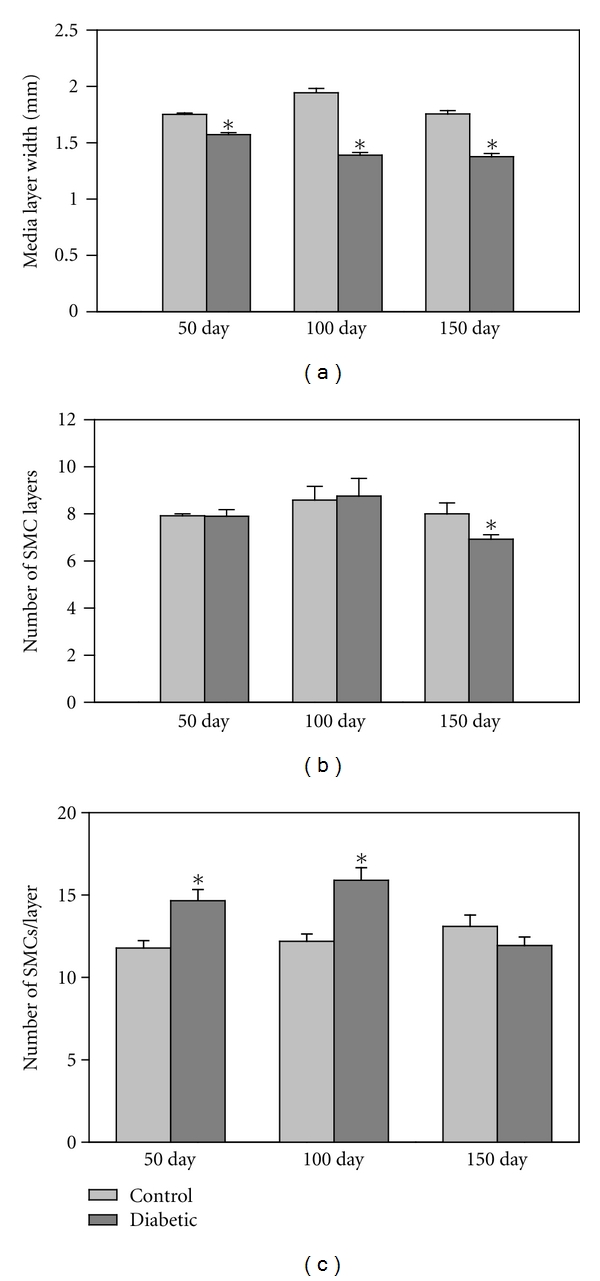
Tunica media characteristics in aorta. (a) The media width was significantly reduced in the diabetic aortas when compared to the control aortas at all three time points (*P* ≪ 0.001, *n* = 150–165 measurements/group from 6 rats/group). (b) There was no difference in the number of SMC layers until 150 days at which time the diabetic aorta showed a reduction in the number of layers compared to the respective control group (*P* < 0.03) and the other diabetic groups (*P* < 0.01). Asterisks denote statistical differences between control and diabetic pairs (*n* = 10–12 measurements/group). (c) The number of SMCs per media layer was assayed with H&E-stained sections. There was a significant increase in the SMC number/layer in the diabetic rat aortas at 50 and 100 days (*P* < 0.005) but returned to control levels at 150 days of diabetes (*n* = 10–12 measurements/group).

H&E-stained sections illustrated differences between diabetic and control vessels in the intima-media width, and the width and number of SMC layers (Figures 2 and 3). The width of the aortic wall was statistically greater in the control rats compared to the diabetic animals at every time point (Table 2, *n* = 30 measurements/group from 6 rats/group). At the same time, the tunica media width from diabetic rats was significantly less than from the control rats at all three time points (*P* < 0.05) (Figure 3(a)). Approximately 30–37% of the total area of the aortic wall was comprised of SMC, and that percentage did not change with time for either experimental groups (Table 2, *n* = 25 measurements/group from 4 rats/group). In the femoral artery, the SMC comprised 45–67% of the wall area with the diabetic group larger only at the 50 day time point (Table 3, *n* = 5/group from 3 rats/group). The number of SMC layers in the aorta remained relatively constant at the 50 and 100 days of diabetes, but was significantly lower in the 150 day diabetic group compared to the corresponding controls (*P* < 0.05) and the other diabetic groups (*P* < 0.05, Figure 3(b)). The layer numbers were not significantly different between any of the three control groups (Figure 3(b)). Consistent with that result, there was no difference in the SMC area compared to the ECM area for the aorta (Table 2). In general, the femoral arteries had higher values for the SMC/ECM area ratios than the aortic values, as expected. The only difference noted between the control and diabetic animals from the femoral arteries was at the 50 days and that was reversed at the 100 and 150 day time points (Table 3).

 The number of SMCs per layer, indicative of cellular proliferation, was significantly higher at 50 and 100 days in the diabetic rats when compared to their respective control groups (*P* < 0.005) (Figure 3(c)). However at the 150 day time point, there was a decline in the number of SMCs per layer in the diabetic group that was significantly lower than the other diabetic groups (*P* < 0.005), and therefore not significantly different from the 150 day control samples. The number of SMC per layer did not change significantly in any of the control groups (Figure 3(c)).

### 3.4. SMC Ultrastructure

**Figure 4:**
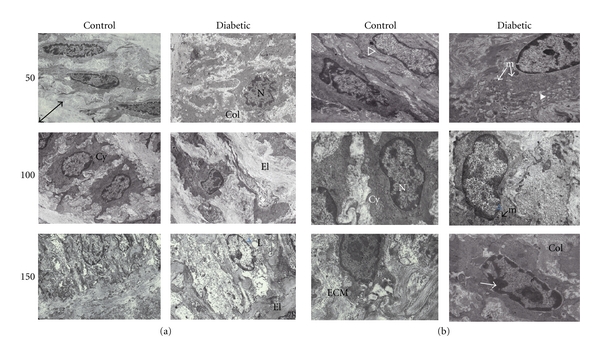
Examples of electron micrographs of aorta. (a) EM micrographs (8,700x magnification) showed a distinct organization of SMCs within each layer, sandwiched by elastic bands (El). The arrowed line in the 50 day control image illustrates a typical elastic band width measurement. In the diabetic aorta, patches of SMC disorganization were evident. Nuclei (N) of cells from diabetic animals often appeared to have a different spatial orientation with invaginated membranes (50 days). The cytoplasm (Cy) was more spread out with finger-like projections in the diabetic samples. The matrix surrounding the SMC contained a mixture of collagen (Col) and elastin (El). (b) Micrographs (21,600x magnification) show SMC nuclei (N) surrounded by cytoplasm (Cy) either rich in cytoskeletal fibers (clear ⊳) as seen in the control samples, or in secretory vesicles (white ⊳) as noted in the diabetic samples. Mitochondria (m) had an increased incidence of swelling or disruption in the diabetic samples. The nuclei contained heterochromatin (→) lining the inner nuclear envelope.

In control animals, aortic SMCs formed a distinct and nicely organized arrangement (Figure 4(a)) with an ECM mix of collagen and elastin surrounding each cell. The SMC layers were sandwiched between elastic bands. At higher magnification, SMCs from control animals displayed elongated nuclei surrounded by cytoplasm rich in cytoskeletal components with mitochondria that were intact (Figure 4(b)). The nuclei contained heterochromatin lining the inner nuclear envelope. Some nuclei had deep invaginations. Visually, the SMC from diabetic rats exhibited changes in the cytoplasm such as increased secretory vesicles and rough ER (Figure 4(b)). The SMCs within each layer were less organized in vessels from diabetic animals, presenting a fragmented appearance when compared to the control samples.

**Figure 5:**
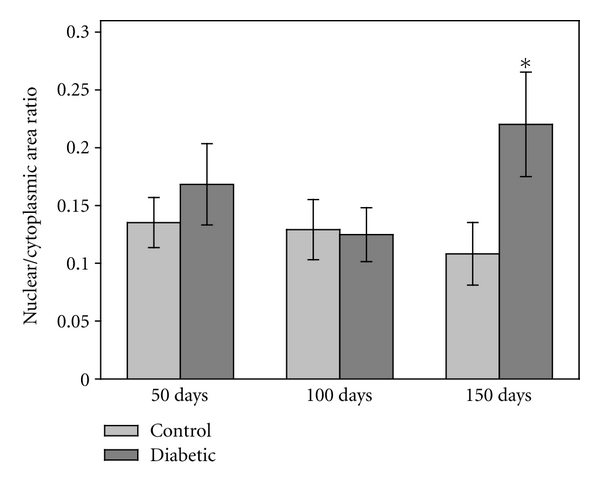
Nuclear/cytoplasmic cross-sectional area. Evaluation of the electron micrographs demonstrated that the ratio of nuclear/cytoplasm area remained constant at the 50- and 100-day time points. At 150 days, the ratio significantly increased in the SMC from the diabetic rats (*P* < 0.05). The change in ratio was due to a corresponding increase in nuclear area and a subsequent reduction in cytoplasm area (*n* = 26–32 measurements/group for 50 day rats, *n* = 15 measurements/group for 100 and 150 day rats).

 An increase in the ratio of nuclear to cytoplasmic area has been used as a basic indicator of cellular hypertrophy [21]. Based on this calculation, there were no statistically significant changes suggestive of SMC hypertrophy at 50 or 100 days of diabetes (Figure 5), and the same was true of the SMCs within the femoral artery (Table 3). With 150 days of diabetes, the ratio of nuclear/cytoplasmic area in the diabetic aorta was significantly higher than samples from control animals (*P* < 0.05) while it remained unchanged in the femoral artery (Table 3).

 While the shape of the nucleus appeared to change with extended diabetes (Figure 4, 150 days), certain morphological features within the nucleus were not affected. The amount of heterochromatin lining the inner nuclear membrane (measured as % of nuclear area) was not different in the aortic samples with diabetes (Table 2), but there was less heterochromatin in the diabetic femoral samples at 50 days (Table 3). The number of nuclear invaginations (defined as nuclear membrane infoldings of at least 0.4 *μ*m in depth) was not different between control and diabetic groups at any time point and from either aortic or femoral samples (Tables 2 and 3, resp.).

**Figure 6:**
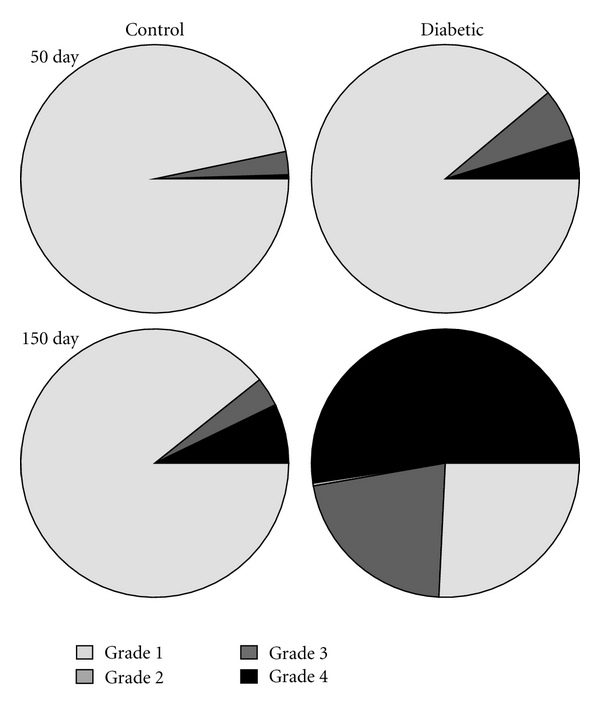
Mitochondrial membrane disruption in aorta. Mitochondria were graded on the basis of membrane intactness with grade 1 as a completely intact organelle. Grade 2 indicated disruption of the outer mitochondrial membrane. Grade 3 indicated disruption of the inner mitochondrial membrane, and grade 4 meant that both membranes were disrupted. In the SMCs from control animals, the mitochondria were predominantly intact throughout the course of the study. In contrast, in the SMCs from the diabetic animals there were more grade 2–4 mitochondria present at day 50 and the percentage of disrupted mitochondria grew over the 150 day diabetes duration until over half of the mitochondria determined to be grade 3-4. Grade 3 measurements were more rare and shown as a thin sliver in the diabetic 150-day figure (*n* = 821 and 1046 mitochondria for 50-day control and diabetic respectively; *n* = 418 and 425 for 100-day control and diabetic groups; *n* = 330 and 446 for 150-day control and diabetic groups).

With 150 days of diabetes, significant portions of mitochondria were swollen or disrupted giving a vacuolated appearance to the SMC cytoplasm (Figure 4(b)). The state of the mitochondrial membranes was significantly disrupted in all diabetic groups compared to the corresponding nondiabetic groups (*P* < 0.005). The percentage of intact mitochondria was based on previously published methods of quality assessment [18]. There was progressive disruption in the mitochondria of tissue samples from control animals (possibly due to aging) from 3% at 50 days to 19% at 150 days (Figure 6). Likewise, there was a progressive disruption of the mitochondria from diabetic aortas from 11% at 50 days to 60% at 150 days. Analysis of the femoral artery samples indicated that there was no diabetes-induced disruption of the mitochondria in any of the groups (*n* = 800–1000 mitochondria at each time point).

### 3.5. Lipid Droplet Content

**Figure 7:**
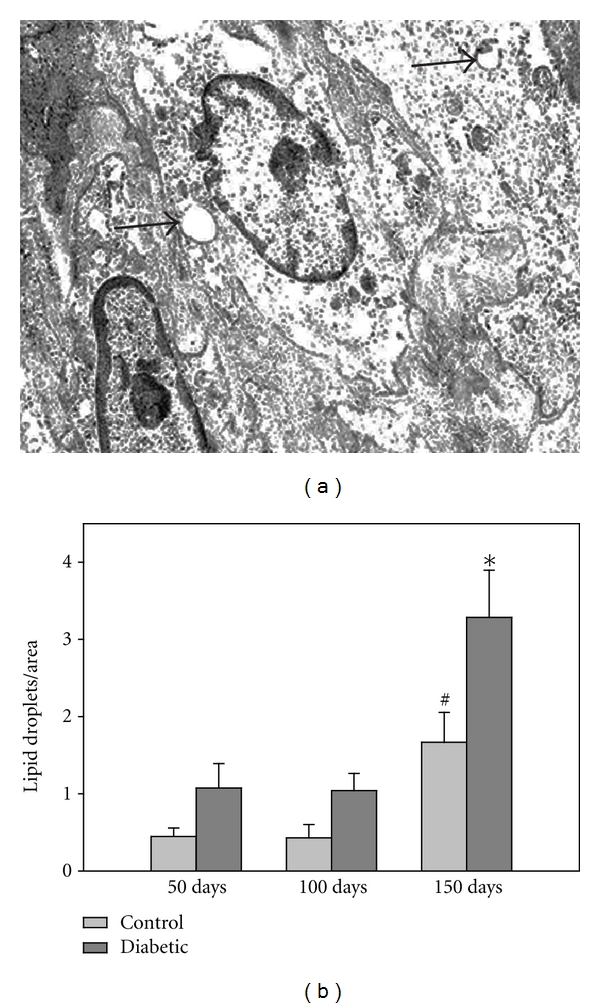
Lipid droplets in aorta. (a) Arrows indicate lipid droplets in a diabetic aortic sample. (b) The number of lipid droplets within the aortic SMC increased in the 150 day diabetic and control animals (*P < 0.01). The lipid incidence tended to increase with age in the control rats, becoming significant at 150 days (^#^ *P* < 0.02). (*n* = 47–56 measurements/group from 50 day rats, *n* = 14–24 measurements/group from 100 day rats, and *n* = 21–24 measurements/group from 150 day rats).

Although there were trends toward more lipid droplets in the aortas of the 50 and 100 day diabetic rats compared to the respective controls, the difference was not statistically significant until 150 days (*P* < 0.01) (Figure 7). The femoral artery samples yielded similar results with only the 150 day groups showing a statistically significant difference (Table 3). In control vessels, the lipid droplet incidence also tended to increase with age becoming significantly greater at 150 days (*P* < 0.05) in contrast to the other two time points.

### 3.6. Extracellular Matrix

**Figure 8:**
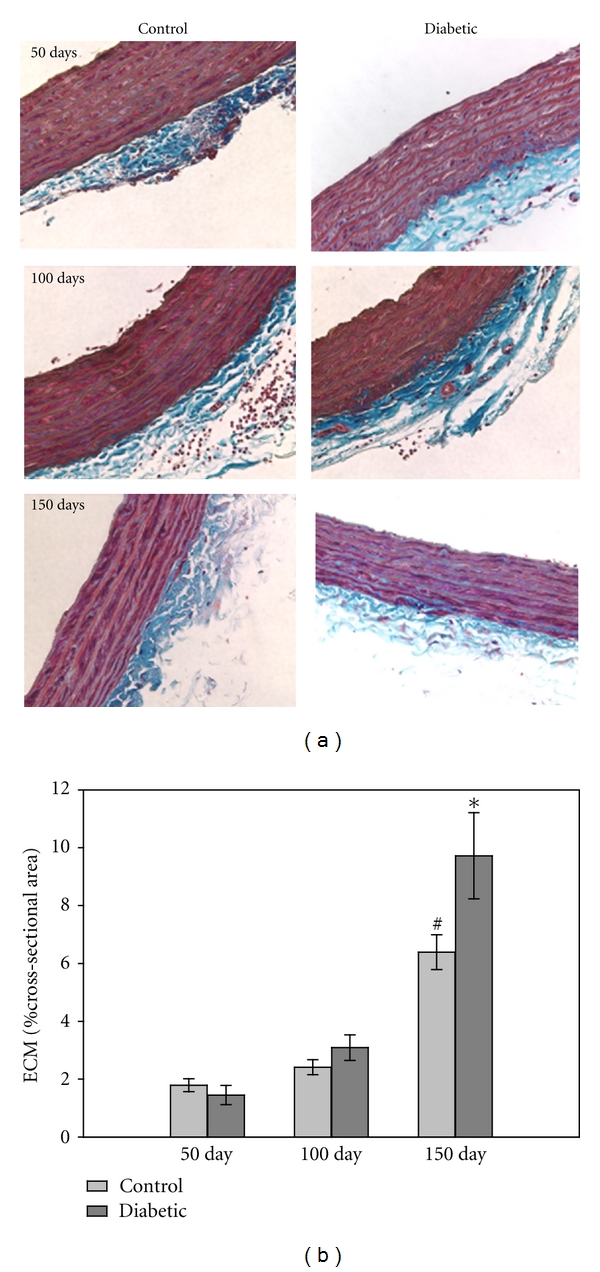
ECM in aorta. (a) Masson's trichrome-stained imaged demonstrated that the amount of ECM (blue staining) increased with time in both control and diabetic aortas (magnification = 40x). (b) The increase in ECM fractional area was significant for the diabetic group at 150 days (*P* < 0.001) and the matched control group (^#^ *P* < 0.001) (*n* = 10–12/group for each time point).

Perhaps the most dramatic changes in the diabetic vessels were noted in the ECM. Masson's trichrome-stained sections demonstrated differences in ECM amount found within the SMC layers and in the adventitia (Figure 8(a)). When compared to controls, the ECM fractional area found within the tunica media, measured by grouped color analysis, was significantly higher (*P* < 0.05) in the 150 day diabetic compared to the 50-day group (Figure 8(b)).

**Figure 9:**
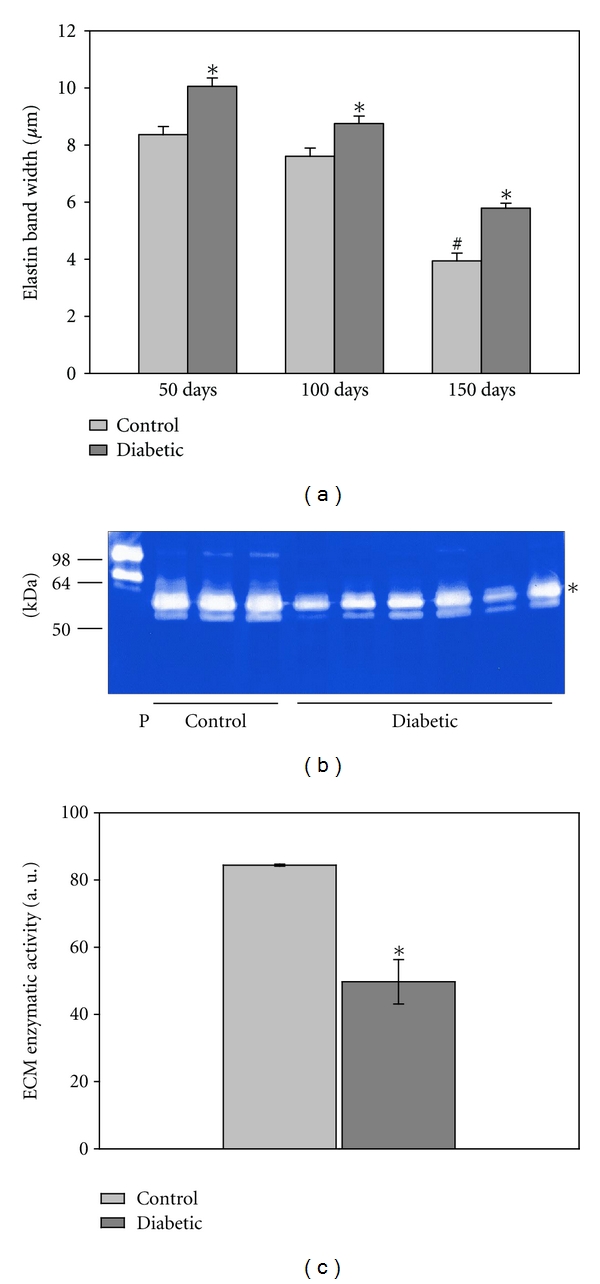
Extracellular matrix. (a) The elastic band width, measured from EM sections, was significantly higher in all diabetic samples (*P < 0.001) although it followed the control pattern of decline with age (^#^ *P* < 0.05; *n* = 80 measurements from 6 rats/group for 50 day, *n* = 100 measurements from 3 rats/group for 100 day, and *n* = 60 measurements from 3 rats/group for 150 day). (b) A representative zymography is shown. Zymography of control and diabetic aortic proteins revealed decreased MMP activity with diabetes. Bacterial collagenase (5 ng/lane) was used as a positive control (P) for zymography. Molecular weight markers are indicated to the left. (c) Zymogram quantification revealed a statistically significant decline in MMP activity. (*n* = 3/group). The band included in the analysis is the most intense, as indicated with the asterisk.

 Changes in the area of elastic components were assessed by measuring the elastic component width (Figure 9(a)), illustrating increased elastin band width in the aortic samples from diabetic animals at each time point (*P* < 0.005). These differences were also observed in the femoral artery (Table 3). With age the elastic band area fell in both the control and diabetic groups of the aortic and femoral samples. The ratio of the SMC to ECM areas was not statistically different in the diabetic groups compared to vessels from the control animals (Tables 2 and 3). In the femoral artery, the 50-day diabetic group had greater SMC/ECM area ratios than controls (*P* < 0.01) and this trend reversed by 100 days. In fact, there was more ECM area as a percentage of the total vessel area in the 150-day diabetic aorta, but not in the other samples tested (Table 2, *n* = 11–13 measurements/group).

 In order to determine whether the diabetes-induced increase in ECM levels noted in Figure 9(a) was due to greater collagen production or decreased collagen breakdown, activity levels of matrix metalloproteases (MMPs), enzymes degrading collagen, and other ECM components, were measured from the 50-day group.  Figure 9(b) illustrates a representative image of a zymography gel. There was a 41% decrease in the MMP activity in aortic extracts from the diabetic rats, compared to respective controls (Figure 9(c)).

## 4. Discussion

In this study, structural changes were identified in the large vessels of a rat model of type 1 diabetes. The structural changes were present before any changes in resting blood pressure or arterial elastance were detected. Some of the most dramatic changes were found in the width of the media tunica layer of the vessel walls, in the percentage of intact mitochondria, deposition of intracellular lipid droplets, and in the extracellular matrix.

Without diabetes, the structure of the vessels changed very little over the 100 days monitored. The SMC layer width increased over time, while the elastic layer width decreased, thus keeping the media width constant. The quality of mitochondria in the SMCs from control animals progressively worsened over time, suggesting that the changes noted in the control and diabetic animals were not an artifact of tissue fixation, but associated with the aging and the disease process. Most of the changes observed in ultrastructure as a function of time were in the extracellular matrix, particularly collagen. While the elastin steadily declined, the collagen content increased over time; an alteration known to increase vessel stiffness. In humans the age-related decline in the mechanical properties of the aorta and resistance arteries includes central aortic compliance and pulse wave velocities [22]. The aortic stiffness has been strongly associated with age even when adjusted for differences in resting blood pressure [23–25].

Vascular changes associated with diabetes have been described as accelerated aging [24]. In this study, diabetes significantly altered mitochondrial morphology in the vascular SMC. Similar changes have been reported previously in vascular endothelial cells from a mouse model of type 1 diabetes [26]. It has been suggested that one of the underlying mechanisms responsible for diabetes-induced vascular disease is uncoupled mitochondria, with increased electron leak and the generation of reactive oxygen species [27, 28]. 

Measurements of stiffness and vascular compliance are important measures for people with type 1 diabetes. Recently, structural changes in the carotid arteries of type 1 diabetics without overt vascular disease were examined according to age and the duration of diabetes [9]. The researchers found that intima-media thickness was one of the first parameters to change in the diabetic population. In fact, increased intima-media thickening has been reported in children and adolescent with short-term diabetes [29]. This correlates with our finding of intima-media thickening even prior to changes in systemic vascular elastance.

Aortic stiffness in people with type 1 diabetes without hypertension correlated with a decline in brain white matter, as measured by MRI. In fact aortic stiffness predicted white matter atrophy [30]. In women with type 1 diabetes, vascular changes in the aorta cause significant functional disorders [31]. Large vessel stiffness was highly correlated with the other complications of type 1 diabetes including nephropathy, retinopathy, and neuropathy [32]. Several factors are important in predicting the advancement of aortic dysfunction in people with diabetes. Age is one of the most important, as it is an independent predictor of stiffness and pulse wave velocity [9]. In addition, the duration of diabetes predicts the intima-media cross-sectional area and systolic blood pressure [9]. However, if a person with type 1 diabetes maintains tight glycemic control, arterial elastic properties can be improved [33].

Most of the diabetic characteristics increased with the duration of diabetes in our model. However, a few reversed that trend including the number of SMC SMC/media layer, which showed a statistical difference at the 50- and 100-day mark, but not at 150 days. Caution should be used when evaluating these changes as only 3 animals per group survived 150 days with uncontrolled diabetes. While the effect size was large for most of the diabetic-induced changes in vessel characteristics, a lack of statistical difference in a small sample size may not be predictive of a larger population. One interesting correlation was the drop in blood glucose levels in the diabetic rats at 150 days. It is possible that certain morphological features are exquisitely sensitive to the blood glucose level, thus explaining why some parameters were not statistically different at the late time point. An additional explanation for the plateau effect may be in the general physiological condition of the surviving diabetic rats. Twenty-one weeks (150 days) is an extensive period of time for an animal to survive with uncontrolled diabetes. Finally, it is possible that there is truly a cellular plateau or cellular adaptation at which no further morphological changes can occur.

This study assists in defining the diabetic time-dependent alterations in macrovascular structure and cellular function. At the structural level we determined that with the progression of diabetes, ECM content becomes higher in the media while smooth muscle cell layers and number of smooth muscle cells per layer decline. Ultrastructural changes show increased cellular hypertrophy and alterations in mitochondria morphology with diabetes. There was an overall age-dependent increase in media lipid droplets, but with a much higher incidence in the diabetic animals and an age-dependent change in SMC nuclear morphology not related to diabetes. How any one of these observations contributes to the development of diabetic macrovascular disease will keep the field of diabetes vascular research busy for a very long time.

## Figures and Tables

**Table 1:** Mean rat blood glucose levels: mean nonfasting blood glucose levels show elevated readings for the diabetic groups throughout the duration of the study (*n* = 16 control and 19 diabetic animals).

Blood glucose	Control	Diabetic
50 day	112.7 ± 4.7	553.7 ± 13.9*
100 day	101.1 ± 4.3	513.5 ± 23.8*
150 day	109.5 ± 3.7	444.1 ± 10.9*

*indicates *P* < 0.05.

**Table 2:** Diabetes-induced changes in the aorta: vessel (intima-media) width was measured as shown in Figure 2 and was significantly less with diabetes for each time point. There were no statistical differences between groups in the total percentage of the vessel wall comprised by SMCs and the SMC/ECM area (as presented in Figure 3), the area containing heterochromatin, and the invaginations within the nuclear envelope. The differences in the amount of ECM were not significantly different until the 150-day time point.

	Duration of diabetes					
Experimental group	50 days		100 days		150 days	
	Control	Diabetic	Control	Diabetic	Control	Diabetic
Vessel width (mm)	1.75 ± 0.01	1.57 ± 0.02 *P* < 0.001	1.94 ± 0.03	1.39 ± 0.04 *P* < 0.001	1.76 ± 0.02	1.00 ± 0.03 *P* < 0.001
SMC area (percent of vessel wall, including adventitia)	30.2 ± 1.5	33.3 ± 2.3	35.7 ± 1.9	34.6 ± 2.1	36.9 ± 2.8	36.9 ± 2.8
Heterochromatin (% of nuclear area)	42 ± 2	48 ± 3	46 ± 3	43 ± 3	39 ± 4	38 ± 2
Invaginations per nuclear envelope	1.10 ± 0.22	1.11 ± 0.20	1.08 ± 0.30	0.55 ± 0.17	0.89 ± 0.27	0.82 ± 0.22
SMC area/ECM area (ratio)	0.60 ± 0.04	0.76 ± 0.07	0.99 ± 0.21	0.67 ± 0.16	0.87 ± 0.16	0.87 ± 0.03
ECM (percent of vessel wall area)	1.79 ± 0.22	1.45 ± 0.33	2.4 ± 0.26	3.09 ± 0.44	6.39 ± 0.60	9.72 ± 1.49 *P* < 0.024

**Table 3:** Diabetes-induced changes in the femoral artery: mimicked the changes in the aorta with the exception of the SMC/ECM area which was statistically higher in the femoral artery of the 50-day diabetic animals, but not in the aorta. Heterochromatin was less in the diabetic samples at 150-days. Lipid droplet density was statistically greater in the diabetic group at 150 days. Finally the elastin band width was greater in the diabetic group throughout the study duration.

	Duration of diabetes					
Experimental group	50 day		100 day		150 day	
	Control	Diabetic	Control	Diabetic	Control	Diabetic
SMC area (percent of vessel wall)	45.0 ± 2.7	62.2 ± 3.3 *P* < 0.04	60.8 ± 10.6	47.5 ± 2.6	67.0 ± 3.9	62.7 ± 5.3
SMC area/ECM area (ratio)	0.86 ± 0.09	1.73 ± 0.24 *P* < 0.009	1.76 ± 0.75	0.93 ± 0.10	2.17 ± 0.30	2.17 ± 0.30
Nuclear/cytoplasmic area (ratio)	0.066 ± 0.029	0.066 ± 0.022	0.132 ± 0.108	0.049 ± 0.014	0.031 ± 0.012	0.0470 ± 0.021
Heterochromatin (% of nuclear area)	40 ± 2	31 ± 3 *P* < 0.05	33 ± 1	37 ± 4	32 ± 4	31 ± 1
Invaginations per nuclear envelope	0.24 ± 0.16	2.17 ± 0.54	1.03 ± 0.20	0.50 ± 0.25	0.79 ± 0.42	3.00 ± 1.06
Lipid Droplets/area	0.8889 ± 0.3889	2.300 ± 0.8825	0.4286 ± 0.1727	1.0417 ± 0.2210	1.6667 ± 0.3885	3.2857 ± 0.6100 *P* < 0.034
Elastin band width	0.9614 ± 0.0336	1.1556 ± 0.0305 *P* < 0.001	0.8764 ± 0.0316	1.0072 ± 0.0302 *P* < 0.01	0.4072 ± 0.0202	0.6662 ± 0.0192 *P* < 0.001
